# Occult Breast Cancer Presenting as Axillary Lymphadenopathy: A Case Report

**DOI:** 10.7759/cureus.107738

**Published:** 2026-04-26

**Authors:** Varvara Pantelidou, Evangelos Perdikakis, Nikolaos Archontis, Demetrios Hatzibougias, Athanasios Permekerlis

**Affiliations:** 1 1st Surgical Department, 424 General Military Hospital, Thessaloniki, GRC; 2 Interventional Radiology Department, 424 General Military Hospital, Thessaloniki, GRC; 3 2nd Surgical Department, 424 General Military Hospital, Thessaloniki, GRC; 4 Department of Pathology, Microdiagnostics Medical S.A., Thessaloniki, GRC

**Keywords:** axillary lymph node dissection, multidisciplinary team, occult breast cancer, primary breast cancer, ultrasound

## Abstract

Occult breast cancer (OBC) is an intriguing entity posing great challenges in diagnosis and treatment. A 58-year-old female was referred to the breast department of our hospital with a metastatic left axillary node from breast carcinoma. Mammography, ultrasound, and MRI of the breast did not reveal any primary breast tumor. According to the decision of the multidisciplinary team, the patient underwent axillary lymph node dissection (ALND), followed by adjuvant chemotherapy, radiotherapy (RT), and hormone therapy. At present, three years later, the patient is free of disease. So far, various treatment options for OBC have been described. Although ALND combined with RT seems to be a safe oncological approach, limited evidence and individualized management in this radiologically occult disease are of paramount importance. Furthermore, awareness of this clinical entity is important for early diagnosis and appropriate management.

## Introduction

Occult breast cancer (OBC) is a rare entity, which represents 0.3-1% of all breast cancer diagnoses [1]. The first description of OBC was made by Halsted et al. in 1907. It is considered a metastatic carcinoma of breast origin without any clinical or radiologic evidence of a primary breast tumor. So far, the development of OBC remains unclear. It has been hypothesized that OBC arises from ectopic breast tissue in the axillary lymph nodes [2]. It has also been suggested that lymph node metastasis occurs sub-clinically due to activation of angiogenesis in the axilla, instead of the breast [3].

Common radiologic armamentarium, such as mammography, ultrasound, and MRI, which are standard diagnostic tools, fail to detect the primary tumor, and the most common clinical manifestation is metastatic axillary lymph nodes, which are sometimes palpable [4]. However, axillary metastasis without a known primary could also come from other sites (lung, melanoma, lymphoma, etc.), which is why proving breast origin is of paramount importance. Diagnosis is made through biopsy of the abnormal lymph node and additional immunohistochemistry (IHC) testing, such as GATA binding protein 3 (GATA‑3), estrogen receptor (ER), and human epidermal growth factor receptor 2 (HER2), which helps establish breast origin [5]. Despite recent advances in breast imaging, the diagnosis of OBC remains challenging [6]. At present, there are few retrospective studies regarding OBC, and therefore various treatment options have been described. Axillary lymph node dissection (ALND) combined with radiotherapy (RT) seems to be an acceptable and safe oncologic approach. However, the dilemma of whether to perform breast surgery and especially mastectomy still exists in the literature [7].

Herein, we report a rare case of a 58-year-old female with OBC who presented with axillary lymphadenopathy.

## Case presentation

**Figure 1:**
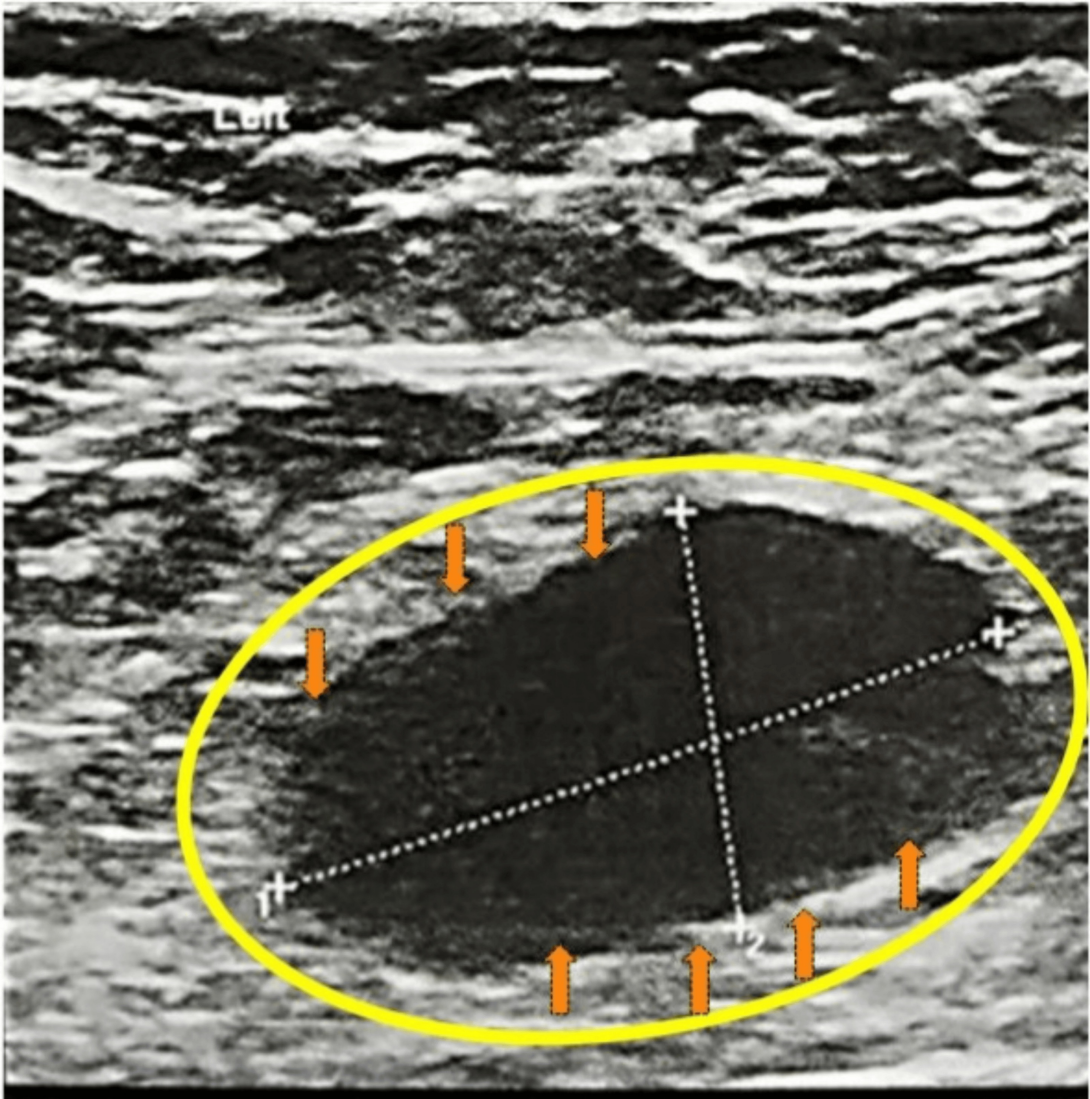
Ultrasound image. The ultrasound image shows an abnormal axillary lymph node (demonstrated with arrows and a yellow circle), which has a lobulated shape and loss of normal echotexture with disappearance of the normal fatty hilum.

**Figure 2:**
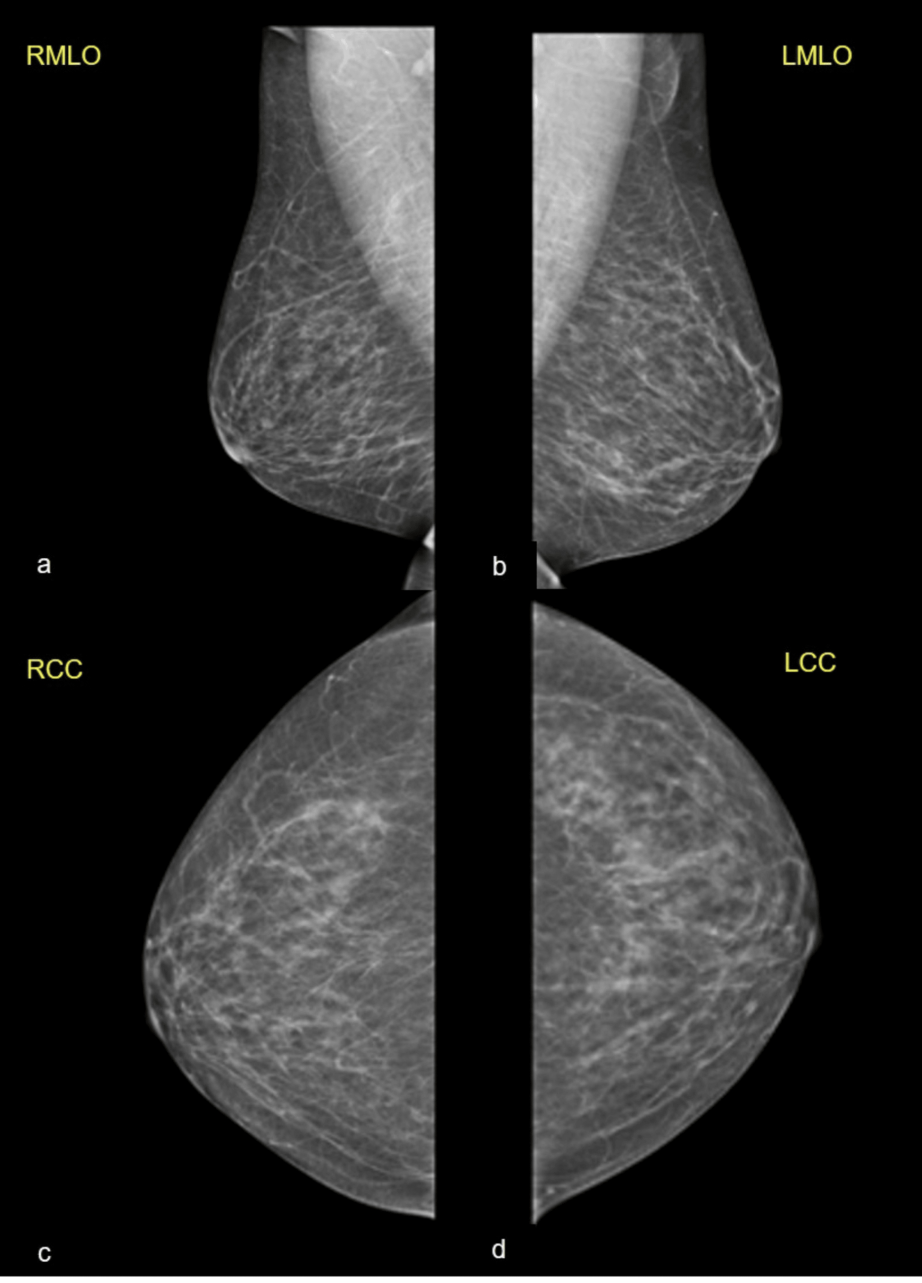
Mammographic images. Normal mammogram showing dense fibroglandular tissue without focal masses or suspicious microcalcifications.

**Figure 3:**
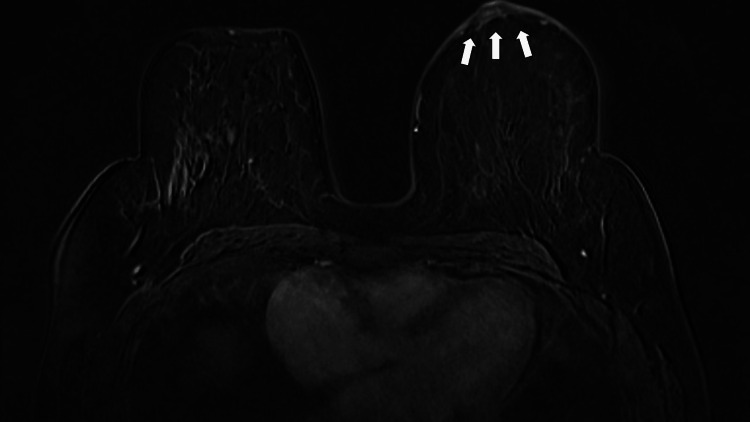
Breast magnetic resonance imaging. The digital subtraction T1-weighted contrast-enhanced MRI shows slight uptake in the left subareolar area (arrows).

**Figure 4:**
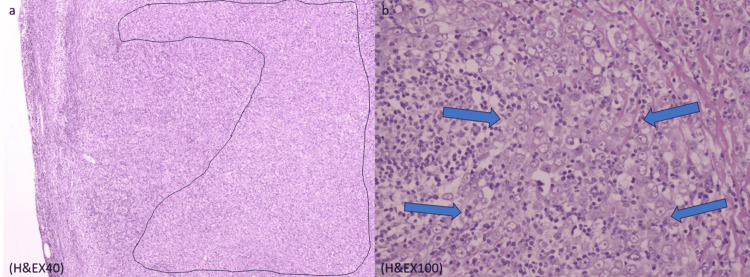
Preoperative histopathologic images. Preoperative histopathologic images (a-b) reveal lymph node parenchyma infiltrated by neoplastic cells (H&E x40 and H&E x100 magnification views). (a) The circled area indicates part of a lymph node that is diffusely infiltrated by neoplastic cells. (b) Blue arrows indicate big neoplastic cells with hypochromic enlarged nuclei, pronounced nucleoli, and eosinophilic cytoplasm. Scale bar: 50.000 μm.

**Figure 5:**
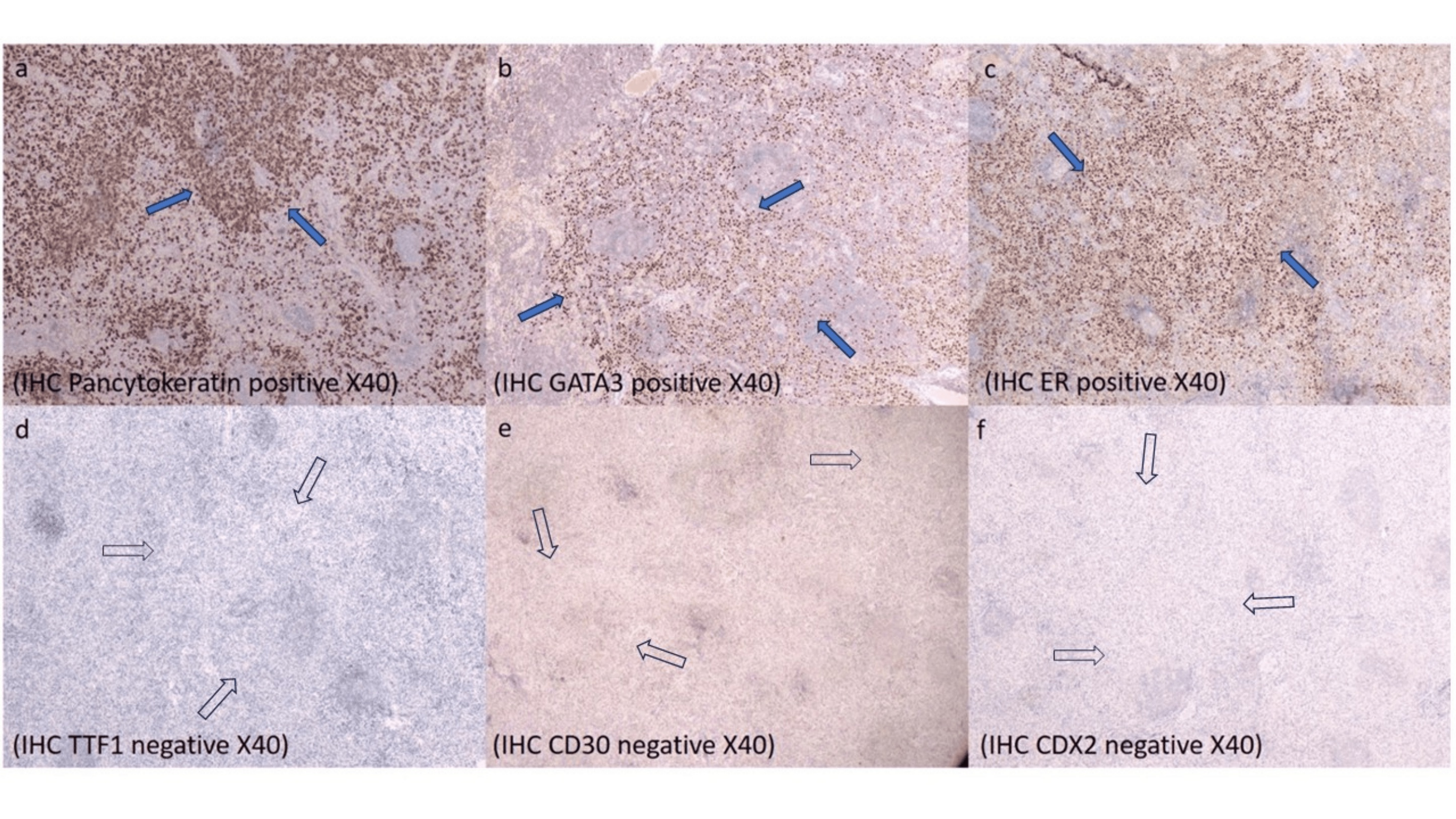
Preoperative immunohistochemistry testing. Preoperative immunohistochemistry testing (a-f: pancytokeratin, GATA-3, and ER positive, and TTF1, CD30, and CDX2 negative stains) demonstrated that the neoplastic cells were of breast origin. (a): Pancytokeratin; (b): GATA-3; (c): ER. Brown staining indicates nuclear positivity of neoplastic cells. Blue arrows indicate the positive neoplastic cells. (d): TTF-1l (e): CD30l (f): CDX2. White arrows indicate a negative neoplastic cell population. Scale bar: 50.000 μm. IHC: immunohistochemistry; GATA-3: GATA binding protein 3; ER: estrogen receptor; TTF1: thyroid transcription factor-1; CDX2: caudal-type homeobox-2.

A 58-year-old post-menopausal female had her annual screening mammogram and ultrasound, which revealed axillary lymphadenopathy without any abnormal findings from the breast. Ultrasound showed an abnormal, enlarged axillary lymph node with cortical thickening and loss of the fatty hilum, measuring 2 x 1.4 cm (Figure 1). Mammography was reported normal: "Normal mammographic findings showing dense fibroglandular tissue without focal masses or suspicious microcalcifications" (Figure 2). Magnetic resonance (MR) mammographic examination showed an abnormally enlarged and lobulated lymph node (diameter: 2 cm), classified as Breast Imaging Reporting and Data System (BI-RADS) category 4, with slight enhancement of the areola (Figure 3). Core needle biopsy (CNB) of the lymph node was performed two weeks after initial imaging, and histologic and immunohistochemical testing revealed diffuse infiltration of numerous neoplastic cells (Figure 4), which were positive for pancytokeratin, GATA-3, and ER, and negative for thyroid transcription factor-1 (TTF-1), CD30, and caudal-type homeobox-2 (CDX2) receptors (Figure 5). Findings were indicative of a metastatic breast carcinoma.

**Figure 6:**
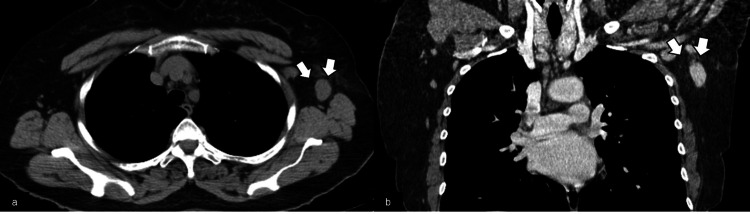
Multidetector computed tomography (MDCT). The axial (a) and the coronal (b) MDCT demonstrate axillary lymphadenopathy in the left (arrows).

**Figure 7:**
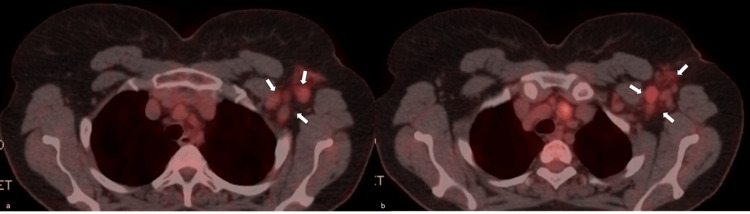
Positron emission tomography-computed tomography (PET-CT). The axial (a-b) PET-CT images show axillary lymph nodes (arrows) on the left axilla that demonstrate increased uptake of 18F-FDG (SUVmax = 3.4). Mediastinal blood pool SUV = 2.6. SUVmax: maximum standardized uptake value; SUV: standardized uptake value; 18F-FDG: 18F-fluorodeoxyglucose.

Afterwards, she was referred to the breast department of our hospital. She had a clear past medical history and did not receive any drugs. She reported no family history of breast or ovarian cancer. Upon clinical examination, she had no palpable lymph nodes in the left axilla and no other clinical findings in either breast. The patient underwent a bone scintigraphy, which was normal, a thoracic and abdominal computed tomography (CT), which verified abnormal left axillary nodes (Figure 6), and a positron emission tomography/computed tomography (PET/CT) scan (Figure 7), which showed an increased uptake of 18F-fluorodeoxyglucose (18F-FDG) (maximum standardized uptake value (SUVmax) = 3.4). A second ultrasound and core biopsy from multiple sites of the left areolar area were performed, four weeks after initial imaging, which showed atypical lobular hyperplasia (ALH). Three core biopsies from the left subareolar area showed foci of ALH involving two of the three core samples. E-cadherin immunostain showed loss of expression.

**Figure 8:**
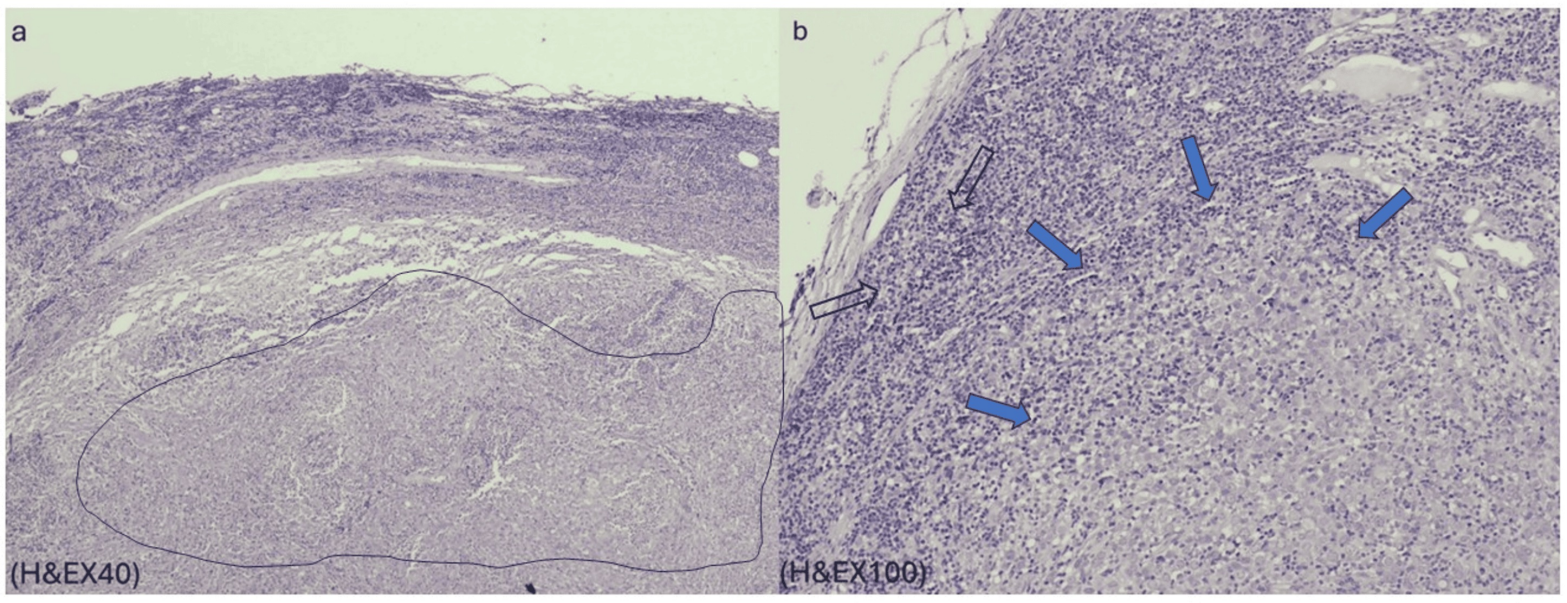
Postoperative histopathologic images. Postoperative histopathologic images (a-b) show a lymph node with neoplastic cells (H&E x40 and H&E x100 magnification views). (a) Circulated area indicates diffuse infiltration of the lymph node by neoplastic cells. (b) Blue arrows indicate clusters of neoplastic cells, with eosinophilic cytoplasm, enlarged nuclei, and prominent nucleoli, even under x100 magnification. White arrows indicate normal lymph node parenchyma. Scale bar: 50.000 μm.

The case was discussed in the multidisciplinary team (MDT) meeting, and an operation was scheduled. She was submitted to an ALND without any breast surgery (BS), six weeks post initial screening. Histopathologic examinations revealed that 11 out of 11 removed lymph nodes were infiltrated by invasive breast carcinoma. There was no extranodal extension of tumor cells. There was no clearly differentiated pattern of the tumor cells, which formed solid neoplastic forms. No ductal or lobular histopathologic pattern was observed (Figure 8).

**Figure 9:**
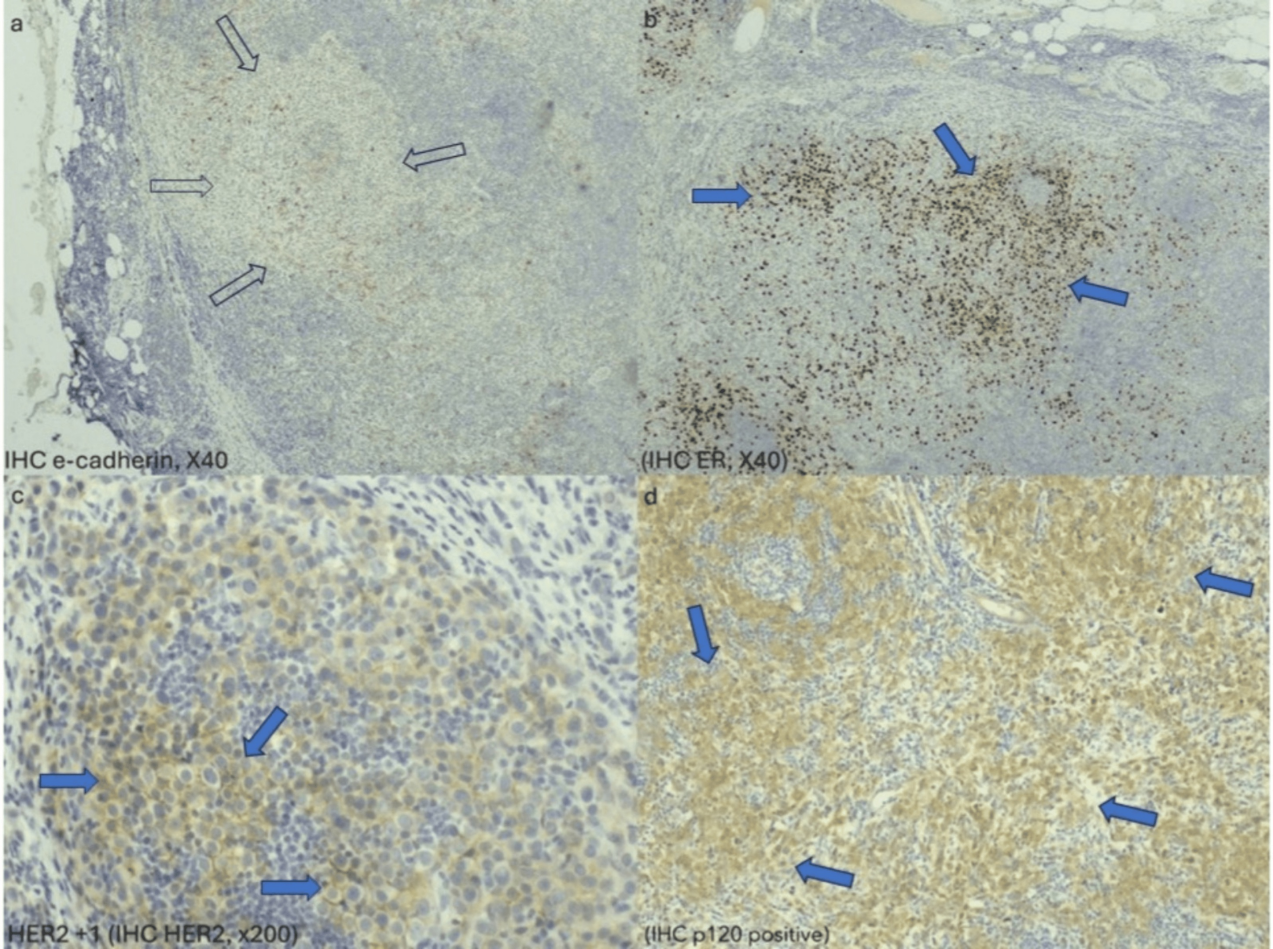
Postoperative immunohistochemistry testing. Postoperative immunohistochemistry testing (a-d: cadherin, ER, HER2, and p120 stains) confirmed that the neoplastic cells were of breast origin. (a) E-cadherin: White arrows indicate the negative neoplastic cell population. (b) ER: Blue arrows indicate brownish nuclear positivity of many neoplastic cells. (c) HER2: Blue arrows indicate brownish membranous positivity of neoplastic cells, stain intensity 1+. (d) p120: Blue arrows indicate cytoplasmic positivity in many neoplastic cells, consistent with breast lobular carcinoma. Scale bar: 50.000 μm. IHC: immunohistochemistry; ER: estrogen receptor; HER2: human epidermal growth factor receptor 2.

IHC testing revealed that the neoplastic cells were positive for GATA-3, ER, and progesterone receptors (PR) and negative for E-cadherin, TTF1, CD30, and CDX2 receptors. Her2 protein was tested with IHC and highlighted membranous positivity in 2% of the tumor cell population with stain intensity 1+ (Figure 9). Therefore, fluorescence in situ hybridization (FISH) Her2 test was not performed. P120 stain highlighted cytoplasmic positivity in numerous neoplastic cells, a finding that is consistent with lobular carcinoma. Of tumor cells, 15% were positive for the Ki67 immunostain. The aforementioned immunophenotype seems to reflect the luminal A subtype. The postoperative course was uneventful, and she was discharged on postoperative day one, with a drain, which was removed 12 days later.

The patient was referred to the MDT for further treatment. The stage of the disease was T0, N3, M0. She received four cycles of epirubicin-cyclophosphamide and four cycles of docetaxel (therapeutic timeline: two to five months post surgery), followed by whole breast radiotherapy (WBRT) six months post surgery and hormone therapy month seven onwards (letrozole 2.5 mg daily).

During the three-year follow-up, the patient did not have any recurrence of the disease or any complication of the ALND (no lymphedema, shoulder dysfunction, or chronic pain, and full range of motion). Follow‑up included clinical examination every six months, annual mammography and breast ultrasound, and symptom‑directed imaging. No recurrence was detected at 36 months. She will continue hormone therapy for five years, in accordance with guidelines.

## Discussion

Since there is no clinical or radiological evidence of a primary breast tumor, the diagnosis of OBC remains a challenge. The first step is a CNB, and it is usually preferred over fine needle aspiration (FNA). Accurate pathologic assessment and immunohistochemistry testing, including ER, PR, and HER2 analysis, is of paramount importance, and in cases of inadequate sample, biopsy has to be repeated, even by surgical means. If the neoplasm has a breast origin and mammography and ultrasound do not reveal the primary tumor, then MRI is indicated. If MRI reveals the primary tumor, then the diagnosis of OBC is excluded [8]. In more than 50% of cases, the undetected primary cancer is found by MRI [9]. PET/CT is not used in the daily clinical routine and is indicated in female patients with radio-dense breasts [6]. In our case, core biopsy was diagnostic of metastatic breast cancer, and the subsequent radiologic workup excluded both the presence of a primary breast tumor and metastatic disease elsewhere.

So far, there is no consensus about the optimal management of OBC. Traditionally, patients with axillary lymph node metastasis of unknown primary origin received mastectomy and ALND. Nowadays, many patients are treated with ALND, followed by WBRT with satisfactory results [8]. According to a recent meta-analysis by Wang et al. [7], the treatment options for OBC described in the literature include ALND or sentinel lymph node biopsy (SLNB) only, ALND with RT, ALND with breast surgery (BS), ALND with RT and BS, and finally RT only. The meta-analysis concluded that breast surgery, modified radical mastectomy (MRM) or breast-conserving surgery (BCS) combined with ALND and followed by RT may improve survival. Some studies do not support that BS improves the prognosis [10,11]. In fact, the histopathologic examination of the final specimen of the breast surgery may reveal a primary breast lesion and therefore the diagnosis of OBC is excluded. Of course, identifying a primary breast lesion improves prognosis [7].

According to recent recommendations of the National Comprehensive Cancer Network (NCCN), patients with T0, N1, M0 disease could be treated either with ALND plus WBRT with or without nodal irradiation or alternatively with mastectomy plus ALND. Patients with T0, N2-3, M0 should be considered for neoadjuvant chemotherapy, trastuzumab, and endocrine therapy. The above recommendations are based on limited evidence originating from a few retrospective studies involving small numbers of patients [8]. So far, there are limited data to support that WBRT to an intact breast provides adequate local control in OBC, compared to mastectomy. However, several retrospective studies have shown that ALND and RT provide similar outcomes compared with mastectomy, in terms of recurrence, distant metastasis, and survival [12].

Since OBC is a metastatic carcinoma in the axilla with no primary breast lesion, lymph node status is the strongest prognostic factor [13]. According to the American Joint Committee on Cancer staging system, a minimum of 10 regional lymph nodes (LNs) is necessary for accurate evaluation and staging in patients with breast cancer. The positive lymph node ratio (PLNR) is the ratio of the number of positive LNs to the number of regional LNs examined. So far, it has been suggested that PLNR is a superior prognostic factor to pathologic node stage in cancer patients [14]. According to a population-based cohort study, PLNR is an independent prognostic factor for OBC. Interestingly, not only PLNR, but also the number of examined lymph nodes (ELNs) is significantly associated with breast-cancer-specific survival (BCSS). Patients with more than 10 ELNs had a better BCSS than those with fewer than 10 ELNs, suggesting that an adequate number of dissected LNs is critical for patient prognosis [15]. The aforementioned data demonstrate the significance of ALND in the staging and prognosis of OBC patients. In our case, ALND defined the accurate stage of the disease, which was T0N3, and the removal of more than 10 lymph nodes may affect prognosis.

In our case, breast cancer presented with an abnormal axillary lymph node detected by ultrasound, and the final diagnosis was confirmed by CNB of the abnormal lymph node. Per the NCCN guidelines [8], clinically node‑positive (N1) disease may be considered for neoadjuvant therapy, but our patient had no palpable nodes, and MDT opted for upfront ALND. Ultrasound and mammography were reported as normal. MRI showed only some minor findings in the areola area, but the core biopsy revealed only ALH. Based on the preoperative findings and the existing evidence, ALND was performed, followed by chemotherapy, WBRT, and endocrine therapy. The stage of the disease was T0, N3, M0. At present, three years after the diagnosis, the patient is free of disease.

## Conclusions

Diagnosis and treatment of OBC demands high clinical suspicion and awareness of this rare entity in cases of axillary lymphadenopathy, in the absence of a primary breast tumor. Although ALND combined with RT seems to be a safe oncological approach, the optimal treatment is individualized. Such cases should be discussed in the MDT, including the breast surgeon, oncologist, radiation oncologist, radiologist, and pathologist, in order to provide the best treatment option and a maximum survival benefit for patients.
